# Multiscale Parallel Algorithm for Early Detection of Tomato Gray Mold in a Complex Natural Environment

**DOI:** 10.3389/fpls.2021.620273

**Published:** 2021-05-11

**Authors:** Xuewei Wang, Jun Liu

**Affiliations:** Shandong Provincial University Laboratory for Protected Horticulture, Blockchain Laboratory of Agricultural Vegetables, Weifang University of Science and Technology, Weifang, China

**Keywords:** multiscale, convolutional neural network, tomato gray mold, object detection, intelligent agriculture, deep learning, plant diseases

## Abstract

Plant disease detection technology is an important part of the intelligent agricultural Internet of Things monitoring system. The real natural environment requires the plant disease detection system to have extremely high real time detection and accuracy. The lightweight network MobileNetv2-YOLOv3 model can meet the real-time detection, but the accuracy is not enough to meet the actual needs. This study proposed a multiscale parallel algorithm MP-YOLOv3 based on the MobileNetv2-YOLOv3 model. The proposed method put forward a multiscale feature fusion method, and an efficient channel attention mechanism was introduced into the detection layer of the network to achieve feature enhancement. The parallel detection algorithm was used to effectively improve the detection performance of multiscale tomato gray mold lesions while ensuring the real-time performance of the algorithm. The experimental results show that the proposed algorithm can accurately and real-time detect multiscale tomato gray mold lesions in a real natural environment. The F1 score and the average precision reached 95.6 and 93.4% on the self-built tomato gray mold detection dataset. The model size was only 16.9 MB, and the detection time of each image was 0.022 s.

## Introduction

Plant diseases are the main cause of food loss in the world’s economy. Food loss from crop infections caused by pathogens such as bacteria, viruses, and fungi is a persistent problem. This situation is further complicated by the fact that disease is more likely to metastasize globally now than ever before. In order to minimize the damage caused by diseases during crop growth, crop prevention is imperative. Traditionally, crop inspections and plant diseases are determined by farmers or experts with some training or experience. This manual method is expensive because it requires continuous monitoring and is not feasible for larger areas.

Tomato is one of the largest vegetable crops planted in China because of its variety, abundant nutrition, and high yield. However, in recent years, gray mold, leaf mold, early blight, late blight, and other common diseases of greenhouse tomato frequently occur, which brings serious threats to the yield and quality of greenhouse tomato. Tomato gray mold is a worldwide infectious disease caused by *Botrytis cinerea*, and it is harmful to the growth of greenhouse tomato. The disease has the characteristics of easy occurrence, rapid spread, strong fungicide resistance, and great economic loss. It has become one of the key factors to inhibit the safe production of greenhouse tomato (Elad and Shtienberg, 1995). In addition to harming tomato, the pathogen can also harm more than 20 crops such as eggplant, pepper, and cucumber. The incidence of tomato gray mold mostly starts from the tip of the leaf and expands inward along the veins in a “V” shape, initially watery, and then yellowish brown, with deep and shallow striate lines on the edges. The boundary between the disease lesion and healthy tissue is clear. Gray mold layer can emerge on the surface of the lesion when the humidity is high, and the region above the pathogenic part can be affected leading to death in severe cases (O’Neill et al., 1997). According to a preliminary field investigation, the tomato yield loss caused by this disease is generally 15–20% and even reaches about 30% in severe cases. The yield loss of tomato gray mold in greenhouse has caused serious losses to national economy and people’s life and has become the main reason that restricts the high efficiency and safe production of vegetables. Therefore, effective control of disease occurrence becomes the key to achieve sustainable development of the tomato industry. At present, chemical agents are mainly used to control tomato gray mold in production. The amount of fungicides used to control gray mold is more than 60% of the amount of fungicides used throughout the planting season. Due to frequent fungicide use, fungicide resistance is prominent and the prevention effect is unsatisfactory (Borges et al., 2015). In the concept of digital agriculture, precise and rational application of fungicides is the direction of agricultural development, and achieving rational application of fungicides is one of the important measures to ensure high yield and safety of vegetables and fruits. It can not only effectively control the occurrence of diseases but also effectively reduce environmental pollution. One of the preconditions for the rational application of fungicides is that information of vegetable growth status must be accurately obtained, among which the key basic technology is to quickly and accurately obtain the types of vegetable diseases and their degree of disease damage. Therefore, the rapid and accurate diagnosis of diseases is an important measure to ensure the high yield and safety of vegetables, which has great practical significance to improve the green, safe, and sustainable production capacity of vegetables.

Existing studies mainly focus on exploring the occurrence of diseases, and there are only a few studies dealing with the early detection of diseases (Nigam and Jain, 2020). Traditional diagnostic methods for plant diseases usually obtain diagnostic results after comprehensive analysis of plant diseases by plant protection workers based on experience and pathological analysis, but these traditional detection methods are inefficient, involve a heavy workload, have poor real-time performance, and are unable to achieve early and rapid diagnosis, often delaying the optimal treatment period of diseases, increasing the dosage of fungicides, and increasing costs and environmental pollution (Martinelli et al., 2015). Because of the complexity and variability of diseases of a large number of plants, even experienced phytopathologists cannot accurately diagnose a specific disease. It is also worth noting that many agricultural regions are difficult to be properly monitored throughout the process (Barbedo, 2013). Early detection of pathogens is essential to reduce disease transmission and promote effective management practices (Sankaran et al., 2010). It is very important to seek a rapid and accurate early detection method.

With the rapid development of facility agriculture in Shouguang City, Shandong Province, China, tomato gray mold has increasingly become a limiting factor affecting tomato production and development, especially in early spring and late autumn tomato cultivation. Using deep neural network to extract features is better than traditional feature extraction methods, and this study will continue to use deep learning methods to detect tomato gray mold lesion objects with different sizes. In this study, images of healthy and infected tomato gray mold leaves in a real natural environment were collected. A multiscale parallel network structure from dense to sparse was proposed based on the universal object detection method YOLO (you only look once). The aims were to establish a rapid and accurate early detection model for tomato gray mold and provide scientific basis for the early diagnosis of tomato gray mold.

## Related Work

### Intelligent Agriculture

Intelligent agriculture, relying on modern information technology, has achieved precision management and visual diagnosis of agricultural production through intelligent perception of the agricultural production environment and data analysis. It is the highest form of agricultural development. Machine vision and its associated emerging technologies hold great potential in intelligent agricultural applications (Tang et al., 2020). Li J. et al. (2020) presented a reliable algorithm based on field RGB-D camera, which can detect and locate fruiting branches of multiple litchi groups accurately and automatically in a large environment. Chen et al. (2020) established a measurement framework of orchard harvesting operation based on multivision technology, and the experimental results show that the proposed adaptive stereo matching strategy has high matching accuracy and stable performance for different sampling depths. Lin et al. (2020) presented a fruit detection method in a natural environment using partial shape matching and probabilistic Hough transform, and experiments on datasets of citrus, tomato, pumpkin, melon, luffa, and mango show that this method is competitive for the detection of most types of fruits in a natural environment, such as green, orange, circular, and noncircular. These studies show that intelligent agriculture based on machine vision and image processing technology has become a key research field in the new agricultural information technology.

### Plant Disease Detection

As an important part of intelligent agriculture, plant disease detection provides a theoretical basis for the scientific formulation of disease control measures and scientific application of drugs. With the application of convolutional neural network in the field of computer vision, the research of plant disease detection has developed rapidly. Convolutional neural network (CNN) is known as a general function simulator, and its ability to fit features is much stronger than that designed based on experience. Initially, researchers simply applied CNN to plant disease detection task, applied network structure for classification to detection task, and only used CNN to extract features from samples. After that, researchers proposed end-to-end plant disease detection models, which could achieve better detection results under a relatively ideal environment (Mohanty et al., 2016; Amara et al., 2017; Durmus et al., 2017; Wang et al., 2017; Aravind et al., 2018; Brahimi et al., 2018; Ferentinos, 2018).

In recent years, the anchor frame-based plant disease detection method has achieved remarkable success, and the most representative one is the Faster region-based CNN (R-CNN-based plant disease detection method). Fuentes et al. (2017) first used Faster R-CNN to locate tomato diseases and pests directly, combined with deep feature extractors such as VGG-Net and ResNet, and the mean average precision (mAP) value reached 85.98% in a dataset containing 5,000 tomato diseases and pests of nine categories. Fuentes et al. (2018) and Fuentes et al. (2019) improved Faster R-CNN on the backbone structure and ROI pooling according to the characteristics of plant diseases and pest detection and the mAP reached 92.5%. Ozguven and Adem (2019) proposed a Faster R-CNN structure for automatic detection of beet leaf spot disease by changing the parameters of the CNN model; 155 images were trained and tested. The results show that the overall correct classification rate of this method is 95.48%. Zhou et al. (2019) presented a fast rice disease detection method based on the fusion of FCM-KM and Faster R-CNN. The application results of 3,010 images showed that the detection accuracy and time of rice blast, bacterial blight, and sheath blight were 96.71%/0.65 s, 97.53%/0.82 s, and 98.26%/0.53 s, respectively. The breakthroughs achieved in the existing studies are amazing. However, the faster R-CNN-based method is a two-stage detection method with a large amount of calculation and is time-consuming.

At present, object detection methods based on deep learning emerge endlessly, and many researchers have improved new methods on plant disease detection to predict the location and class of the lesions. Jiang et al. (2019) proposed the INAR-SSD model, and the test on a self-built apple leaf disease dataset achieved a performance of 78.80% mAP with a high detection speed of 23.13 FPS. Sun et al. (2020) presented an instance detection method improved on the basis of single-shot detector (SSD) to detect maize leaf blight under a complex background. The proposed method combined data preprocessing, feature fusion, feature sharing, disease detection, and other steps. The mAP of the new model is higher (from 71.80 to 91.83%) than that of the original SSD model. The FPS of the new model has also improved (from 24 to 28.4), reaching the standard of real-time detection. Bhatt et al. (2019) presented a method to detect pests and diseases on images captured under uncontrolled conditions in tea gardens. YOLOv3 was used to detect pests and diseases. While ensuring real-time availability of the system, about 86% mAP was achieved with 50% IoU. Singh et al. (2020) concluded that although plant disease detection technology has developed rapidly, the methods can be only effectively used for a restricted number of plants.

### Object Detection Network Structures

The task of plant disease detection is similar to the method of general object detection, and the task of plant disease detection can be seen as the specific application of a general object. As for the classical two-stage universal object detection method, the first stage is responsible for extracting candidate windows, which are the input of the second stage, and the second stage is responsible for the accurate detection task (Ren et al., 2016). The two-stage structures have many parameters and are time-consuming. For example, when performing ROI pooling operation, the candidate box obtained in the first stage needs to be cut out from a high-dimensional feature map. This operation needs a large amount of calculation and requires the aid of parallel computing devices such as GPU.

The emergence of one-stage universal object detection methods has solved this problem. Famous one-stage detection methods include SSD (Liu et al., 2016) and YOLO (Redmon and Farhadi, 2016, 2018; Redmon et al., 2016). Compared with the traditional convolutional neural network, the SSD selects VGG16 as the trunk of the network and adds a feature pyramid network to obtain features from different layers and make predictions. YOLO considers the detection task as a regression problem and uses global information to directly predict the bounding box and category of the object to achieve end-to-end detection of a single CNN network. YOLO can achieve global optimization and greatly improve the detection speed while satisfying higher accuracy. These methods are equivalent to the previous stage of the two stages, eliminating the time-consuming ROI pooling operation and, thus, have an innate speed advantage. The one-stage object detection algorithm directly adds the detection head to the backbone network for classification and regression, uses the whole image as the input of the network, and directly returns the position of the bounding box and the category to which it belongs at the output layer.

In summary, the main difference between the two networks is that the two-stage network needs to first generate a candidate box (proposal) that may contain the lesions, and then further execute the object detection process. In contrast, the one-stage network directly uses the features extracted in the network to predict the location and class of the lesions. In the field of plant diseases and pest detection which emphasizes detection accuracy at this stage, more models based on the two-stage network are used.

### Multiscale Object Detection of Plant Diseases

In actual plant disease detection, multiscale plant disease objects are common in a real natural environment. This study considers how to detect small-scale objects and large-scale objects in the same frame image, such as in an agricultural Internet of Things monitoring scenario, where the proximal plant leaves and the distant plant leaves may have very different scales. In the case of front shooting of the surveillance camera, because some plant disease objects are far away from the camera, the object size is small and it makes the plant disease objects occupy very small pixels in the image. Also, the corresponding area contains less information, which is prone to missed detection, affecting the detection accuracy of the algorithm. Therefore, it is difficult to identify and locate small-scale plant disease targets in the field of target detection.

Because small objects consist of very few pixels and generally only occupy less than 5% of the whole image, it is difficult to extract enough features for CNN. In order to improve the detection performance of small objects, it is usually necessary to combine image super-resolution, large-scale feature map prediction, deep and shallow feature fusion, and other feature enhancement methods (Li D. et al., 2020; Liu and Wang, 2020; Zhao et al., 2020). However, these methods will bring additional parameters and calculation while improving the performance of small object detection, resulting in the reduction of the real-time performance of the algorithm, and it is difficult to deploy to the terminal with a small amount of computation. Therefore, how to improve the performance of small object detection of the algorithm to meet the needs of a real natural environment without introducing too much additional calculation cost to ensure real-time performance is an urgent problem to be solved.

In order to improve the detection ability of multiscale tomato gray mold objects, reduce the missed detection rate, and improve the detection efficiency, MobileNetv2-YOLOv3, which is known for its speed, was selected in this study (Sandler et al., 2018). As a basic detection network, a multiscale parallel tomato gray mold detection algorithm (MP-YOLOv3) with high real-time performance was constructed by combining multiscale pixel feature fusion and efficient channel attention mechanism to enhance the quality of small object features. The contributions of this study are summarized as follows:

(1) A multiscale feature fusion strategy is proposed to fuse feature maps of the skeleton network MobileNetv2 in different scales from high to low, which enhances the small object information carried by feature maps and provides rich semantic information for the prediction layer of the network, thereby effectively improving the small object detection ability of the algorithm.
(2) In order to highlight the useful feature channels and suppress the feature channels with small contribution, we introduce an efficient channel attention module before the detection layer to assign weights to the feature channels according to their importance, which effectively improves the detection performance.
(3) In order to reduce the impact of network complexity on detection speed, a tomato gray mold detection model MP-YOLOv3 with high real time and robustness is proposed, which adopts a parallel processing mode on the architecture and uses a buffer queue between the various functions of object detection to reduce the waiting time in detection.
(4) The model was validated on the self-built tomato gray mold dataset. Compared with existing algorithms, it achieved good results in small-scale tomato gray mold object detection, significantly improved the accuracy of tomato gray mold object detection, reduced the occurrence of missed detection, and could meet the practical application needs.

## Materials

### Dataset Collection

Since there is no published image database of tomato gray mold disease in a real natural environment, 1,000 images of tomato gray mold pathogen in a natural environment were collected from the Internet of Things monitoring video of tomato greenhouse in Shouguang City, Shandong Province, China. Meanwhile, in order to expand the sample dataset, 263 images of tomato gray mold were obtained by the network crawler method. A total of 1,263 images were collected. The images include conditions on cloudy and sunny days, objects such as branches and leaves forming shadows or shelters on the surface of tomato leaves, etc.

### Data Annotation

In image labeling, the minimum outer rectangle of each lesion is labeled with the LabelImg tool^1^ to ensure that there is only one tomato gray mold lesion object in each rectangular labeling frame and as few background pixels as possible. After image annotation, 263 images under different weather and light conditions were selected as the test set, and the remaining 1,000 images were used for network training. Details of 263 images selected are shown in Table 1.

**TABLE 1:** Detailed information of samples in test images.

Conditions	Independent leaves	Indistinct leaves	Shaded leaves	Occluded leaves
				
Number of images	68	62	74	59
Number of annotated lesions	469	457	516	413

### Data Enhancement

When training deep learning model, the more and comprehensive the training data, the stronger the recognition ability (Theodoridis, 2015). Therefore, to enrich the image training dataset, better extract image features, and avoid overfitting, this study uses a variety of methods to enhance the dataset. Due to uncertain factors such as illumination direction and weather, the illumination conditions during image acquisition are very complex. In order to improve the generalization ability of the training model, the original image is processed by eight methods: brightness enhancement and attenuation, color enhancement and attenuation, contrast enhancement and attenuation, and sharpness enhancement and attenuation (Bloice et al., 2017). After image amplification, the original annotation is still valid. Nine thousand images after image enhancement were used for training and parameter optimization validation of subsequent improved network. One thousand images were randomly selected from 9,000 images as the validation set, and the remaining 8,000 images were used as the training set. There was no overlap between the training set and the test set.

## Methods

Considering the real-time requirement of tomato gray mold detection in a real scene, we chose MobileNetv2-YOLOv3 as the basic detection network. However, due to the insufficient accuracy caused by its focus on efficient convolution operation, we combined multiscale feature fusion and efficient channel attention to improve it. This section will give the detailed design methods of the multiscale feature fusion module and the high-efficiency channel attention module and propose the flow of parallel structure.

**FIGURE 1:**
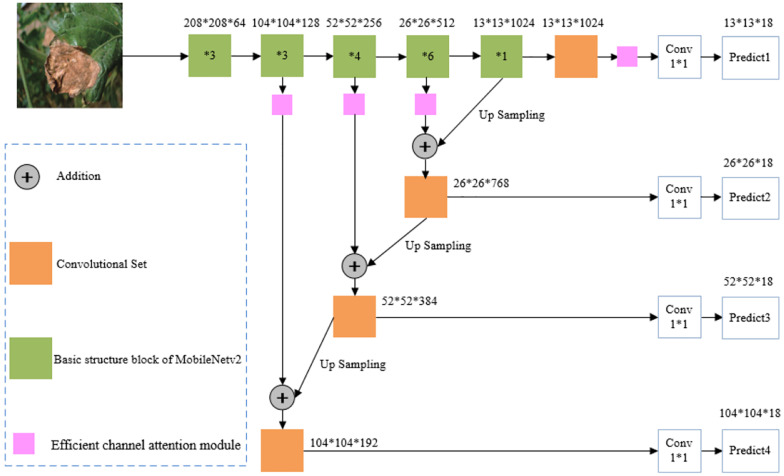
Overall framework of a multiscale parallel algorithm for the early detection of tomato gray mold in a complex natural environment.

The overall framework of the multiscale parallel tomato gray mold early detection algorithm is shown in Figure 1. The algorithm is divided into two parts. The first part extracts the object features through MobileNetv2, and the second part detects tomato gray mold objects through the object prediction part of YOLOv3. First, the image resolution is adjusted to 416 × 416 and it is inputted into the MobileNetv2 network to extract features, and the fused feature map is obtained by multiscale feature fusion. Second, these feature maps are enhanced by the efficient channel attention module, and the weight of feature channels is assigned according to their importance. Third, through MobileNetv2, a 13 × 13 × 1,024-dimensional tensor is obtained, and through a 1 × 1 convolutional kernel for convolution operation, a S × S × 18-dimensional tensor is obtained. Finally, this tensor is used to predict the location of the tomato gray mold.

### Multiscale Feature Fusion Module

In tomato gray mold object detection, due to the distance between the tomato leaf and the camera, the size of the tomato gray mold object presented on the image is also different. The size of the last layer of the feature layer is only 13 × 13, which is 1/32 of the original input image, which makes the feature layer lose some feature information of smaller objects. In deep neural networks, the higher the layers, the smaller the size of the feature map, and the richer the semantic information contained. The lower feature layer has greater resolution and retains more details in the original image, which is conducive to determining the location of the object. To simultaneously utilize the detailed information in the shallow feature map extracted from the MobileNetv2 skeleton network and the semantic information in the deep feature map, a multiscale feature fusion module is proposed in this study. The specific approach is to improve the network’s ability to detect small-size tomato gray mold objects by fusing high-level features with low-level features and predicting them on multiple-scale feature maps. The feature map after up-sampling is combined with the feature map with the size of 26 × 26 in the convolution process as the basis for the second prediction. Then the feature map with the size of 52 × 52 and 104 × 104 is obtained in this way for the third and fourth prediction, respectively, as shown in Figure 1.

### Efficient Channel Attention Module

**FIGURE 2:**
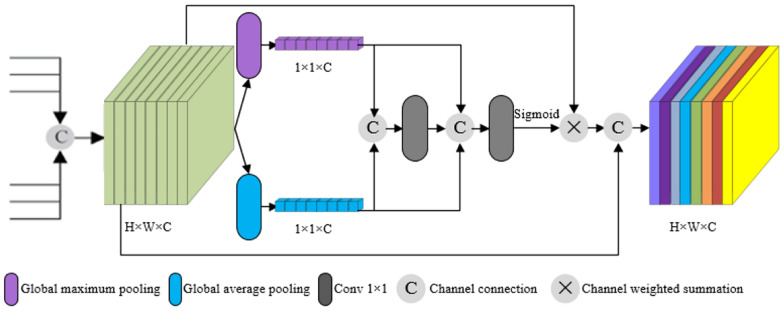
Efficient channel attention module.

When generating fusion feature maps, our algorithm uses the method of channel splicing between the feature maps obtained from the up-sampling and the feature maps extracted from the skeleton network, resulting in large differences in the information carried by each channel fusion feature map. The importance of different channels of fusion features is different for the scale detection, so it is necessary to introduce efficient channel attention module after multiscale merging, so that the model can learn the importance of different channel features. In order to selectively highlight the effective feature channels and suppress the feature channels with small contribution, we detect the feature channels with different scales by assigning weight to the feature channels according to the importance degree through the efficient channel attention module, which effectively improves the detection performance. The principle is shown in Figure 2.

As the spatial characteristics of different channels should have a certain correlation, if a channel has a high correlation with its adjacent channels, it means that the feature contains more subject features. We adopt an efficient channel attention module that can learn the feature correlation between channels. In the efficient channel attention module, in order to highlight the feature correlation between channels, the feature channel dimension is compressed. The original feature channel *H*×*W*×*C* is transformed to 1×1×*C* by global pooling, and global features in channel dimension are obtained. One-dimensional convolution with convolution kernel size *k* is used to extract and integrate information between each channel and its *k* neighborhood channels to obtain correlation parameters *L*_*i*_ between channels.

(1) $$L_{i}=\sum_{j=1}^{k}\alpha^{j}\,C_{i}^{j},C_{i}^{j}\in \varphi_{i}^{k}$$

In the abovementioned formula, α^*j*^ represents one-dimensional convolution kernel parameters, and $\varphi_{i}^{k}$ represents *k* neighborhood channels of feature channel *C*_*i*_. The larger the *L*_*i*_, the stronger the correlation between feature channels *C*_*i*_ and $\varphi_{i}^{k}$, that is, the more useful information *C*_*i*_ contains. To make the network focus on the feature channels with more useful information, ω_*i*_ is denoted as the weight of the feature channel, that is, the activation value of each channel obtained by *L*_*i*_ passing through sigmoid function.

(2) $$\omega_{i}=\sigma \,\left(L_{i}\right)$$

In the abovementioned formula, σ represents sigmoid activation function. The weighted output feature channel is obtained by multiplying the weight with the original channel feature value. The weighted output feature channel is conducive to highlighting the key features of the object and weakening the nonimportant features.

As shown in Figure 1, the efficient channel attention module was added to the proposed model. Feature maps close to the proposed model’s prediction layers have a higher predictive impact, taking into account that more and ineffective computation would be added if efficient channel attention modules were used for all convolutional layers. Therefore, the convolution and addition layers preceding the prediction layers in the model served as inputs to the efficient channel attention module. Using the efficient channel attention module, high weights are assigned to tomato gray mold disease features in the convolutional feature map and low weights to the natural background. The final output prediction layer assigns more weight to the image information of interest, and the information in each channel will contain more accurate and more information on tomato gray mold disease characteristics. Thus, the detection rate is effectively improved, and the small object lesions which are confused by local occlusion and natural background can be easily missed.

### Parallel Network Structure

**FIGURE 3:**
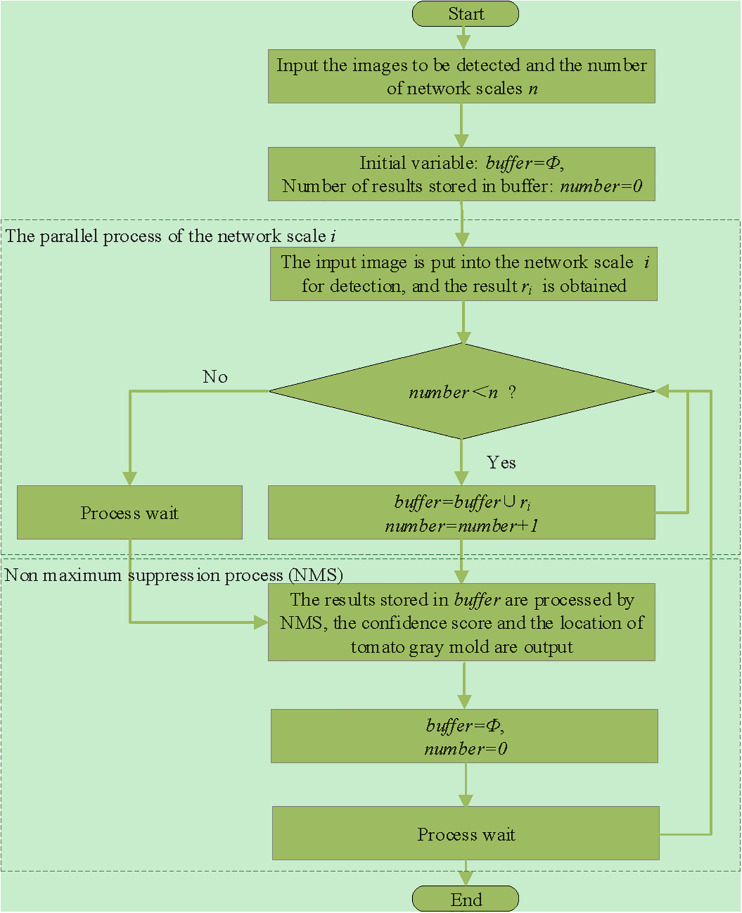
Flowchart of the parallel detection algorithm.

A serial multiscale network will increase the time overhead. To ensure the real-time performance of detection, the improved model uses parallel detection algorithm. The flowchart of the algorithm is shown in Figure 3. The multiscale network changes from serial to parallel operation, and the time overhead is reduced from the time of detection of multiple-scale networks to the time of detection of a single-scale network. It ensures that the time overhead of the model will not increase substantially while improving the detection performance.

### Superiority of the Proposed Algorithm MP-YOLOv3

To present the superiority of the proposed algorithm MP-YOLOv3, the algorithm is compared with Tiny-YOLOv3, MobileNetv2-YOLOv3, MobileNetv2-SSD, and Faster R-CNN regarding their advantages and disadvantages of their specific features. The comparison is shown in Table 2.

**TABLE 2:** Comparison of the characteristics of different algorithms.

Algorithms	Network structure	Backbone network	Feature extraction capability
The proposed method	One-stage	MobileNetv2	Multiscale feature extraction
Tiny-YOLOv3	One-stage	7-layer conv + Max	The ability of deep feature extraction is poor.
MobileNetv2-YOLOv3	One-stage	MobileNetv2	The ability of deep feature extraction is poor.
MobileNetv2-SSD	One-stage	MobileNetv2	The ability of deep feature extraction is poor.
Faster R-CNN	Two-stage	ResNet	The accuracy outperforms some of the one-stage detectors, but the speed is low.

It can be seen from Table 2 that the proposed algorithms are improved by combining multiscale feature fusion and efficient channel attention on the basis of MobileNetv2-YOLOv3. With the addition of very few parameters and little impact on the speed, a high-precision real-time tomato gray mold detection algorithm is constructed.

## Experimental Design

### Experimental Operation Environment

In this experiment, under Ubuntu 16.04 operating system, Caffe deep learning framework was built on i7-7700HQCPU (16 GB memory) and NVIDIA GTX 1070 GPU (8 GB memory) hardware platform, and Python language programming was used to realize the training and testing of the tomato gray mold object detection network model.

### Model Training

In this study, a stochastic gradient descent method was used to train the network in an end-to-end joint manner. In order to improve the training efficiency, during network training, the network parameters were initially initialized with the pretraining model on ImageNet (Deng et al., 2009), so that good initial values of the model were achieved to reduce the training time cost and speed up model convergence. The initial learning rate was set to 0.001, the weight attenuation rate was set to 0.0005, the momentum factor was set to 0.9, and the verification period was set to 5,000, that is, the network tests the accuracy of the training model on the verification set every 5,000 iterations, and the training was stopped when the model accuracy rate reaches convergence. The maximum number of iterations was set to 50,000. Training ended when the loss dropped to around 1. The training model was saved after the training, and the model was verified with the test set. The final output of the network was the identified object and its probability of being the object of tomato gray mold, and the result only retained the region with a probability value greater than 0.8.

### Evaluating Indicator

In this study, both recall rate and accuracy rate should be considered in the process of object detection of tomato gray mold, so F1 value and AP value were used to evaluate the recognition results.

(3) $$F\,1=\frac{2\,P\,R}{P+R}$$

(4) $$P=\frac{T\,P}{T\,P+F\,P}$$

(5) $$R=\frac{T\,P}{T\,P+F\,N}$$

(6) $$A\,P=\frac{\sum P}{N}$$

In the abovementioned formula, *P* represents precision, *R* represents recall rate, TP (true positive) represents the number of tomato gray mold objects that the algorithm can accurately detect, FP (false positive) indicates the number of background misidentified as tomato gray mold objects, FN (false negative) indicates the number of unrecognized tomato gray mold objects, and *N* is the total number of images.

## Experimental Results and Analysis

### Detection Results of Tomato Gray Mold

**FIGURE 4:**
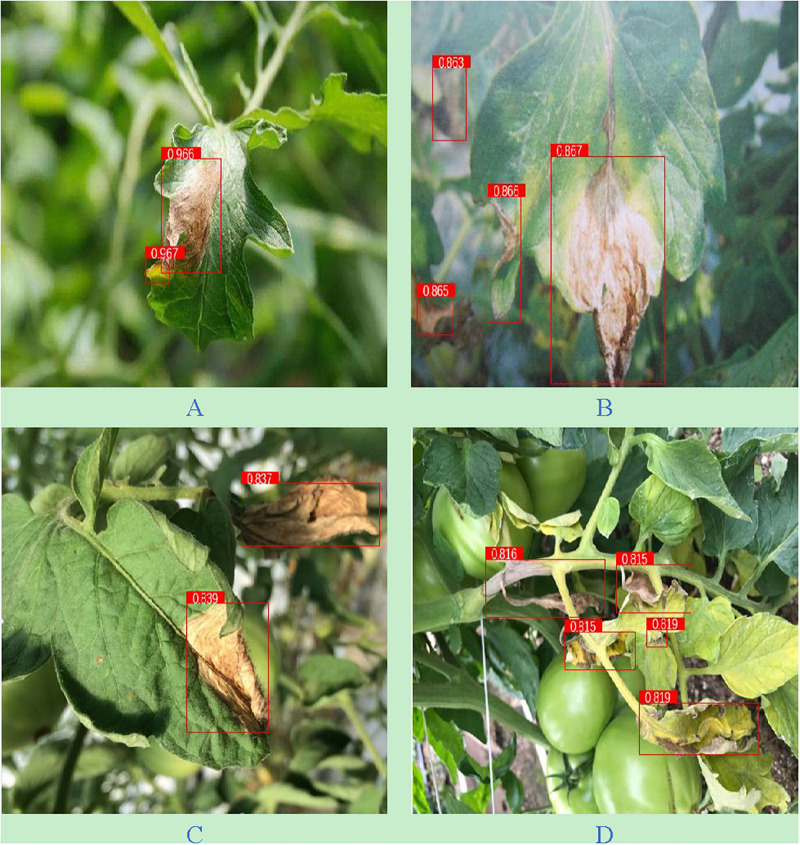
The effect diagram of the detection method in this paper. **(A)** Independent leaves; **(B)** indistinct leaves; **(C)** shaded leaves; **(D)** occluded leaves.

In order to verify the performance of MP-YOLOv3 proposed in this study, the identification results of the network on 263 test sets were further analyzed. There were 1,855 tomato gray mold objects in 263 test sets. The number of objects identified by this method was 1,689, of which 1,578 were tomato gray mold objects. The recall rate, accuracy, and misidentification rate of this method were 85.07, 93.43, and 6.57%, respectively. Examples of the identification results and specific identification results of this research method are shown in Figure 4 and Table 3, respectively.

**TABLE 3:** Detailed detection results of tomato gray mold.

Conditions	Independent leaves	Indistinct leaves	Shaded leaves	Occluded leaves	Total
Number of objects correctly identified	453	396	432	337	1,578
Number of annotated lesions	469	457	516	413	1,855
Recall rate	96.59%	86.65%	83.72%	81.60%	85.07%

From Table 3 and Figure 4, it can be seen that the detection effect is the best in the sparse independent leaf scenario with a recall rate of 96.59%. The blurred indistinct tomato gray mold object can be accurately identified. This method can correctly identify 86.65% of the indistinct objects in the image. This method can effectively identify the tomato gray mold object with a shaded surface, and the recognition recall rate is 83.72%. In addition, this method is also applicable when the leaves are occluded, and its recognition recall rate is 81.60%. According to Figure 4, MP-YOLOv3 also has a high detection accuracy for small-size lesions, which proves that multiscale detection has a good detection effect for objects with different sizes. Through the above analysis, it can be concluded that despite the phenomenon of misidentification and missed identification, the method in this study can accurately detect the tomato gray mold object in the image.

### Analysis of Ablation Experiments

To examine the detection effects of the two improvement modules in MP-YOLOv3, benchmarked on the previous network, the multiscale feature fusion module and the efficient channel attention module were considered as two experimental variables using the dataset built from this study to conduct a series of ablation experiments. Among them, “√” means joining the module and “ × ” means not joining it. The results are shown in Table 4.

**TABLE 4:** Detection effects of ablation experiments.

Strategies	Multiscale feature fusion module	Efficient channel attention module	F1 score/%	Average precision/%
1	×	×	87.9	85.3
2	√	×	91.7	89.6
3	×	√	93.2	91.1
4	√	√	95.6	93.4

As can be seen from Table 4, the two improved modules proposed in this study could both enhance the detection efficacy of tomato gray mold, and the combination of the two modules had the best detection efficacy, which verified the rationality of the model designed in this study. The improvement of the multiscale feature fusion module enriches the feature information of small objects in feature maps. The improvement of the efficient channel attention module enables the feature maps of network output to more efficiently characterize objects.

### Comparison of Different Detection Methods

To verify the detection performance of MP-YOLOv3, the algorithm is compared with Tiny-YOLOv3, MobileNetv2-YOLOv3, MobileNetv2-SSD, Faster R-CNN, and other algorithms. The experimental results are shown in Table 5.

**TABLE 5:** Comparison of detection results using different algorithms.

Algorithms	F1 score/%	Average precision/%	Single image detection time/second (s)
The proposed method	95.6	93.4	0.022
Tiny-YOLOv3	86.8	84.1	0.023
MobileNetv2-YOLOv3	87.9	85.3	0.022
MobileNetv2-SSD	88.5	86.6	0.035
Faster R-CNN	89.9	87.8	0.126

It can be seen from Table 5 that the detection effect of the algorithm in this study is the best among the compared advanced algorithms, and the detection average precision reaches 93.4%, which is 8.1% higher than the AP of MobileNetv2-YOLOv3. By improving the feature extraction network of MobileNetv2-YOLOv3, the number of layers of the network is deepened and the extracted features are more detailed, which ensures that the model improves the detection accuracy at the same time. The number of model parameters did not increase, the size of the model was only 16.9 MB, and the detection time of each image was 0.022 s, which achieved a good detection effect.

## Conclusion and Future Directions

### Conclusions

(1) The method proposed in this study can identify the tomato gray mold object from images with complex background, and it is expected to be applied in tomato growth information monitoring and tomato disease automated inspection. Compared with the traditional method of disease detection, this method is more challenging. On the one hand, the object of tomato gray mold at the early stage of growth is smaller; on the other hand, the object of tomato gray mold at this time is very similar to the background color, resulting in the use of traditional methods which cannot effectively and accurately identify the lesions in the image. Deep learning theory makes early detection of tomato gray mold possible. It can automatically extract image features and is an effective detection method.
(2) The proposed method uses the multiscale feature fusion module and the efficient channel attention module to fuse the features of different scales and effectively solve the problem of insufficient semantic information of low-level features and improve the detection effect of the model on multiscale tomato gray mold objects. The experimental results show that the proposed algorithm has certain advantages over other existing algorithms and solves the problems of multiscale change, occlusion, and poor detection of small-size objects, which can improve the accuracy of object detection while ensuring a small amount of calculation.
(3) The model has excellent performance in practical application and can adapt to a complex natural environment. It lays a research foundation for subsequent disease object positioning and spraying pesticides on demand, reduces the use of chemical pesticides, and has important significance for protecting farmland ecology.

### Future Directions

Crop disease detection is one of the key problems to solve automation in agricultural fields, and object detection is also one of the most difficult tasks of computer vision for a long time. In this study, the task of tomato gray mold object detection was studied, innovative algorithms were proposed, and some progress was made. However, there are still some problems that deserve further study.

(1) In this study, an early detection model of tomato gray mold disease was proposed, and future work needs to further solve the problem of missed detection of under extreme shooting angle to achieve accurate early diagnosis of tomato gray mold disease at different parts under different shooting conditions.
(2) The current work should be transplanted to mobile terminals, such as smartphones, tablet PC, etc., to improve practicability and increase the modern atmosphere. Later in the practical application test, a large number of data will be used to continuously improve the practicability and accuracy of tomato gray mold detection.
(3) Various environmental parameters of greenhouse tomato crops will be collected in real time by the Internet of Things technology, and a tomato gray mold early-warning model will be constructed. Tomato gray mold will be early-warned by analyzing the real-time collected data.
(4) Although this study can achieve excellent detection and recognition results, data-driven deep learning technology requires a large number of samples to support, and it is difficult to obtain sufficient sample size in the field of plant disease monitoring with a wide variety of species. In the future, we will solve this problem from the aspect of small sample disease detection.
(5) Additional care would be necessary with poorly supervised learning when applied to automatic pest detection in our area due to the high cost to deal with labeling work.

## Data Availability Statement

The raw data supporting the conclusions of this article will be made available by the authors, without undue reservation.

## Author Contributions

JL and XW conducted the experiments and data analysis and wrote the manuscript. XW revised the manuscript. Both authors read and approved the manuscript.

## Conflict of Interest

The authors declare that the research was conducted in the absence of any commercial or financial relationships that could be construed as a potential conflict of interest.

## Footnotes

1 https://github.com/tzutalin/labelImg
